# Exploring Saccharomycotina Yeast Ecology Through an Ecological Ontology Framework

**DOI:** 10.1002/yea.3981

**Published:** 2024-09-18

**Authors:** Marie-Claire Harrison, Dana A. Opulente, John F. Wolters, Xing-Xing Shen, Xiaofan Zhou, Marizeth Groenewald, Chris Todd Hittinger, Antonis Rokas, Abigail Leavitt LaBella

**Affiliations:** 1 Department of Biological Sciences, Vanderbilt University, Nashville, Tennessee, USA; 2 Evolutionary Studies Initiative, Vanderbilt University, Nashville, Tennessee, USA; 3 Department of Biology, Villanova University, Villanova, Pennsylvania, USA; 4 Laboratory of Genetics, DOE Great Lakes Bioenergy Research Center, Center for Genomic Science Innovation, Wisconsin Energy Institute, J. F. Crow Institute for the Study of Evolution, University of Wisconsin-Madison, Madison, Wisconsin, USA; 5 Centre for Evolutionary and Organismal Biology, Institute of Insect Sciences, Zhejiang University, Hangzhou, China; 6 Guangdong Province Key Laboratory of Microbial Signals and Disease Control, Integrative Microbiology Research Center, South China Agricultural University, Guangzhou, China; 7 Westerdijk Fungal Biodiversity Institute, Utrecht, The Netherlands; 8 Department of Bioinformatics and Genomics, University of North Carolina at Charlotte, Kannapolis, North Carolina, USA; 9 Center for Computational Intelligence to Predict Health and Environmental Risks (CIPHER), University of North Carolina at Charlotte, Charlotte, North Carolina, USA

**Keywords:** controlled vocabulary, dynamic, formal, isolation environment, macroecology, statistical enrichment

## Abstract

Yeasts in the subphylum Saccharomycotina are found across the globe in disparate ecosystems. A major aim of yeast research is to understand the diversity and evolution of ecological traits, such as carbon metabolic breadth, insect association, and cactophily. This includes studying aspects of ecological traits like genetic architecture or association with other phenotypic traits. Genomic resources in the Saccharomycotina have grown rapidly. Ecological data, however, are still limited for many species, especially those only known from species descriptions where usually only a limited number of strains are studied. Moreover, ecological information is recorded in natural language format limiting high throughput computational analysis. To address these limitations, we developed an ontological framework for the analysis of yeast ecology. A total of 1,088 yeast strains were added to the Ontology of Yeast Environments (OYE) and analyzed in a machine-learning framework to connect genotype to ecology. This framework is flexible and can be extended to additional isolates, species, or environmental sequencing data. Widespread adoption of OYE would greatly aid the study of macroecology in the Saccharomycotina subphylum.

## 1 | Introduction

### 1.1 | The Importance of Yeast Ecology

Over the past 400 million years, the yeasts in the subphylum Saccharomycotina (hereafter referred to as yeasts) spread across Earth, adapting to nearly every biome available (Kurtzman et al. 2011; Shen et al. 2020). The diversity of biotic and abiotic features in these global environments profoundly influenced the diversification and evolution of over 1000 species of yeasts. It led to the evolution of varied genome content, metabolic capabiLities, and phenotypic traits (Shen et al. 2018). Yeasts are now critical components of many different scientific realms: they are used in biotechnology as biofuel and heterologous protein producers (Riley et al. 2016); they play an essential role in the global food supply as plant pathogens, food, and beverage producers (Hittinger et al. 2018), and spoilage yeasts (Loureiro & Querol 1999); and they impact human health as commensal (Suhr and Hallen-Adams 2015) and pathogenic (Bidaud, Chowdhary, and Dannaoui 2018) components of the mycobiome.

The environments in which yeast thrive are as varied as the yeasts themselves. They are predicted to be most commonly found in mixed montane forests in temperate climates. However, yeasts have been sampled directly from the atmosphere, including from clouds (Vaïtilingom et al. 2012). In the aquatic realm, yeasts can be found in very high densities across freshwater, marine, and deep-sea environments (Nagahama 2006). Within the deep-sea, yeasts have been found at deep-sea hydrothermal vents (Keeler et al. 2021), cold seeps (Nagano et al. 2014), and whale falls (Nagano et al. 2020). In the Arctic, yeasts have been isolated from seawater, subglacial ice, and brine puddles on sea ice (Butinar, Strmole, and Gunde-Cimerman 2011). On land, yeasts are found to be associated with abiotic substrates and living or dead organisms. Abiotic environments that host yeasts include soil (Botha 2011), caves (Cunha et al. 2020), and rock surfaces (Selbmann et al. 2014).

Yeasts have also evolved intimate relationships with many different organisms. Yeasts, plants, and insects form complex systems where some or all the partners benefit. This includes the well-known cactus-yeast-*Drosophila* (Goncalves et al. 2023; Starmer and Fogleman 1986) and flower-yeast-beetle systems (Blackwell 2017). Other animals from which yeasts have been isolated include cows (Brejová et al. 2019), horses, chickens, bats, apes, and cats (Kurtzman et al. 2011). Yeasts play a major role in the digestive tracts of animals ranging from insects (Stefanini 2018) to humans (Pérez 2021). In association with plants, yeasts are found on leaves (Sláviková et al. 2007), in plant exudates (Bowles and Lachance 1983), and associated with roots (Sarabia et al. 2017). Yeasts also play a major role in the environment as decomposers of plant matter (Cadete, Lopes, and Rosa 2017). This list is not exhaustive, but it demonstrates the breadth of niches that yeasts inhabit.

Yeasts from these varied habitats exhibit different, likely adaptive traits. Yeasts isolated from cold seeps in the deep sea are adapted to low temperatures (Nagano et al. 2014). Yeasts isolated from mammalian digestive tracts can resist stressors, such as the immune system (Rosenbach et al. 2010). A better understanding of where yeasts reside and their ecological niche breadths will allow us to test hypotheses regarding how their diverse ecological traits evolved, what yeast traits might emerge in the future, and what intrinsic or extrinsic factors have shaped their observed patterns of diversity in species across the yeast subphylum.

Uncovering genetic variants associated with ecological traits of yeast species remains a major challenge. Traditionally, researchers identify a trait and subsequently identify the genetic features that influence it. For example, the beak morphology of Darwin’s finches is associated with variation in bone morphogenic protein 4 (BMP4) (Abzhanov et al. 2004). Identifying genetic contributors to ecological traits in microbes can be challenging due to sampling limitations, unknown genetic backgrounds, and complex phenotype-environment interactions (Brettner et al. 2022), even for well-characterized traits. For example, the ability of yeasts to produce and accumulate ethanol under aerobic conditions (the Crabtree/Warburg Effect) is associated with multiple genetic changes (Postma et al. 1989) and arose approximately 125–150 million years ago (Hagman & Piskur 2015). Did microbial competition lead to this innovation? If so, under what specific conditions or environment did this trait arise? Previous analyses cannot confidently identify the forces shaping this trait due to the evolutionary time scale and lack of information about the ecological niche of extant yeasts (Hagman & Piskur 2015). The known ecological data for Crabtree/Warburg-positive Saccharomycetaceae are highly varied. *Tetrapisispora phaffii* has been isolated once from African soil in the 1960s (Kurtzman et al. 2011). Conversely, *Kluyveromyces marxiaunus* has been isolated from foods, beverages, decaying plant tissue, and insects (Kurtzman et al. 2011). Given this data, we cannot make any clear connections between ecology and the Crabtree/Warburg Effect, let alone its adaptive significance. In other cases, different yeast species may share a trait, but the underlying genetic associations are not the same. For example, while most yeasts utilize the Leloir pathway to metabolize d-galactose, some yeasts appear to utilize an alternative oxidoreductive d-galactose pathway (Harrison et al. 2024). Conversely, many yeasts contain the enzymes necessary to metabolize xylose but are unable to grow on xylose in a laboratory setting (Nalabothu et al. 2023). These features—long evolutionary time scales, limited ecological data, complex genetic traits, and more—make traditional ecological studies difficult.

One approach that addresses some of the issues noted above is “Reverse Ecology,” in which traits and their underlying genetic variation are inferred directly from genomic information (Levy & Borenstein 2012). There are vast genomic resources available in yeasts, from thousands of strains within a species (Peter et al. 2018) to a genome for nearly every known yeast species (Opulente et al. 2024). This latter species-level data set, known as the Y1000+ Project (http://y1000plus.org) data set, provides genomes for 1154 yeast strains from 1051 species and, importantly for reverse ecology, phenotypic and ecological data. Yeast researchers have already begun to interrogate diverse ecological traits and link ecology or habitat with specific yeast traits and underlying genome variation (Cavalieri et al. 2022). Yeasts associated with fruits, fermented substrates, and juices are more likely to have the genomic capability to ferment both glucose and sucrose (Opulente et al. 2018). Cacti-associated yeasts exhibit elevated thermotolerance levels associated with increased evolution rates in cell envelope genes (Goncalves et al. 2023). Yeasts associated with dairy environments have genomic changes related to an increased growth rate on galactose media (LaBella et al. 2021). The data set size allows the utilization of big-data methods, such as machine learning and phylogenomic approaches. However, our current ecological data limit the application of the vast genomic and phenotypic data to address pressing ecological questions such as adaptations to specific environmental niches.

The ecology of yeasts and other microbes can be understood either through direct observation of the organisms in their natural environments or through inference of their potential habitats based on known traits and general ecological principles (Starmer and Lachance 2011). We will focus here on the inference of yeast ecology from their isolation environments. Large-scale databases, such as the Global Biodiversity Information Facility (*GBIF*: The *Global Biodiversity Information Facility* (2024)) and GlobalFungi (Větrovský et al. 2020), provide such data, but they do so for a relatively small number of species. For example, a recent study identified records for 186 yeast species, which amounts to only ~15% of the described species (David et al. 2024). Metagenomic studies are beginning to enable the identification of yeasts from environmental DNA sampling. For example, a study identified the diversity of seven *Saccharomyces* species across elevations and tree habitats (Alsammar et al. 2019). Similarly, a metagenomic study of human cancer samples revealed evidence of 67 Saccharomycotina yeasts but could only identify the species of 23 of these (Narunsky-Haziza et al. 2022). The recent boom in yeast genome sequencing will further allow the identification of more yeasts in metagenomic studies. We anticipate these databases will continue to grow and capture more yeast ecology; capturing this information in digital formats that are consistent across studies will be key for large-scale studies of yeast ecology. In the meantime, there are bountiful opportunities to construct the computational framework for synthesis to leverage the currently available ecological information in novel ways that enable big data analysis.

### 1.2 | Ecological Data and Bio-Ontologies

Ecological data are recorded during the collection of yeasts and documented in species descriptions. According to the current guidelines, species descriptions should include, “A clear statement of the geographic origin and habitat of all isolates” (Lachance 2020). Ideally, this statement would include precise geographic information, detailed substrate description, temperature at the time of collection, and substrate pH. Recorded ecological data, especially historical data, rarely include all these features. In some cases, the data provided are sparse, such as”rotting wood samples were collected in the Sanctuary of Caraça” (Morais et al. 2013). Other descriptions are highly detailed, such as “larvae of *Anastrepha mucronata* (Diptera: Tephritidae) collected from ripe fruit of *Peritassa campestris* (“Bacupari,” Hippocrateaceae)… in the Cerrado ecosystem of the state of Tocantins, Brazil” (Rosa et al. 2006). It is difficult to identify what information might be useful at the time of collection, especially without a universal language to describe environments. Even when detailed information is recorded, it must be re-recorded in a machine-readable format for high-throughput analyses.

Ontologies are an important framework used to transform information described in natural language into a format that allows integration across methods, technologies, and applications (Hastings 2017). Natural language, simply the language used by humans to communicate, is rife with words with multiple meanings and other complexities that make biological interpretations difficult. For example, the word “tree” does not refer to any specific monophyletic group of species—the word tree is used in reference to angiosperms, gymnosperms, and even palms. There is also no universally recognized age at which a sapling should be referred to as a tree or the height at which a shrub transitions to a tree. Therefore, it is reasonable to assume that the word tree represents many different ecological niches. Even species names do not always represent evolutionary relatedness. In Saccharomycotina yeasts, the generic name *Candida* has been used in four different orders, with 32% outside the lineage containing *Candida albicans* (Opulente et al. 2024). Ontologies, like phylogenies, allow us to define precise relationships between biological entities, which allows systematic data analysis and generates a dynamic but controlled vocabulary by which scientists can communicate.

Biological ontologies, also known as bio-ontologies, have become a key resource for scientists. The most popular bio-ontology is the Gene Ontology (GO) (Ashburner et al. 2000). The GO framework consists of three independent ontologies that use dynamic, controlled vocabularies to capture our current knowledge of the molecular functions, cellular components, and biological processes of genes. The success of the GO led to the development of the Open Biomedical Ontologies (OBO) (Smith et al. 2007), which provides best practices, tutorials, and tools for the development of ontologies ranging from Anatomy Ontology (Haendel et al. 2008) to the Zebrafish Phenotype Ontology (Van Slyke et al. 2014). In total, there are 600 ontologies currently listed in the OBO.

Another set of ontologies has been developed specifically to address evolutionary and ecological hypotheses. The Semantics for Comparative Analysis of Trait Evolution (SCATE) was developed to represent complex traits recorded in natural language format as ontologies for evolutionary analysis (Dahdul et al. 2017). This work builds on the success of Phenoscape (http://kb.phenoscape.org), which is an ontology-driven resource aimed at linking phenotypes across fields of biology. It has been used to identify candidate genes associated with phenotypes in fishes (Edmunds et al. 2016). There is also The Environment Ontology which describes environments ranging from ecosystems to planets and even astronomical bodies (Buttigieg et al. 2013). This ontology contains some terms that apply to yeasts, such as “wetland area,” but it cannot account for the many yeasts whose environment is another organism, such as the gut of a beetle. Therefore, the current biological, evolutionary, and ecological ontologies do not fully capture the breadth of yeast environments.

The extensive breadth of environments where yeasts are found necessitated a new ontology. There are bio-ontologies currently available for natural environments (Buttigieg et al. 2013), human anatomy (Haendel et al. 2008), food (Dooley et al. 2018), and plants (Jaiswal et al. 2005). Yeasts are found in all these environments and many more. Moreover, the ecology of some yeasts involves the close relationship between multiple environments. This includes the well-characterized cactus-yeast-*Drosophila* and flower-yeast-beetle systems (Starmer and Lachance 2011). To address the specific challenges of studying yeast ecology, we constructed a new yeast environment ontology using the guiding principles outlined in the Ontology Development 101 (Noy & McGuinness 2001) provided by the team that manages the ontology visualization tool Protégé (Musen & Protege 2015). The ontology was constructed as part of the Y1000+ Project and was used in the flagship publication of the 1154 yeast genomes (Opulente et al. 2024). In this article, the ontology was used to identify overlapping isolation environments between metabolic specialist and generalist yeasts. We will refer to this ontology as the Ontology of Yeast Environments (OYE). Below, we will outline the steps for the construction of the ontology.

## 2 | Methods

### 2.1 | Construction of the OYE

Ontologies are comprised of classes, metadata, relations, and axioms stored in a common file format. We will use a beetle to illustrate these ideas. Classes are the most basic unit and are the hierarchical categories into which observations are placed. We could define “Nitidulid beetle” as a class. Metadata is any information stored within a class and could contain information like a written description. For example, we may include metadata, such as “Nitidulids or sap beetles are insects with defining features such as wing cases.” Relations or modifiers connect classes to each other in the ontology and can include connections, such as “is a part of” to “has function.” We could connect the two classes, “Nitidulid beetle” and “wing-cases,” using a relationship called “is a part of.” Axioms are the rules that constrain classes. All members of the class “Nitidulid beetle” are also members of the class “insect” which makes that an axiom. We will refer to subclasses as any class connected by this type of axiom. This structure allows for flexibility and high-throughput computational analyses. These principles were used in the construction of the OYE

Step 1 was to identify key terms to guide the construction of the ontology. We collected the isolation environment in a natural language form from species descriptions or fungal collections for each strain in our set of 1154 Saccharomycotina yeast strains. In total, we were able to identify information for 1088 yeasts (Supplementary Data s6 from (Opulente et al. 2024)). The information was matched to the strain level to account for the possibility of within-species variation in associations between ecology and genome, such as polymorphisms found in the *GAL*actose metabolism pathway (Hittinger et al. 2010; Lee et al. 2017; Pontes et al. 2024).

**FIGURE 1 |:**
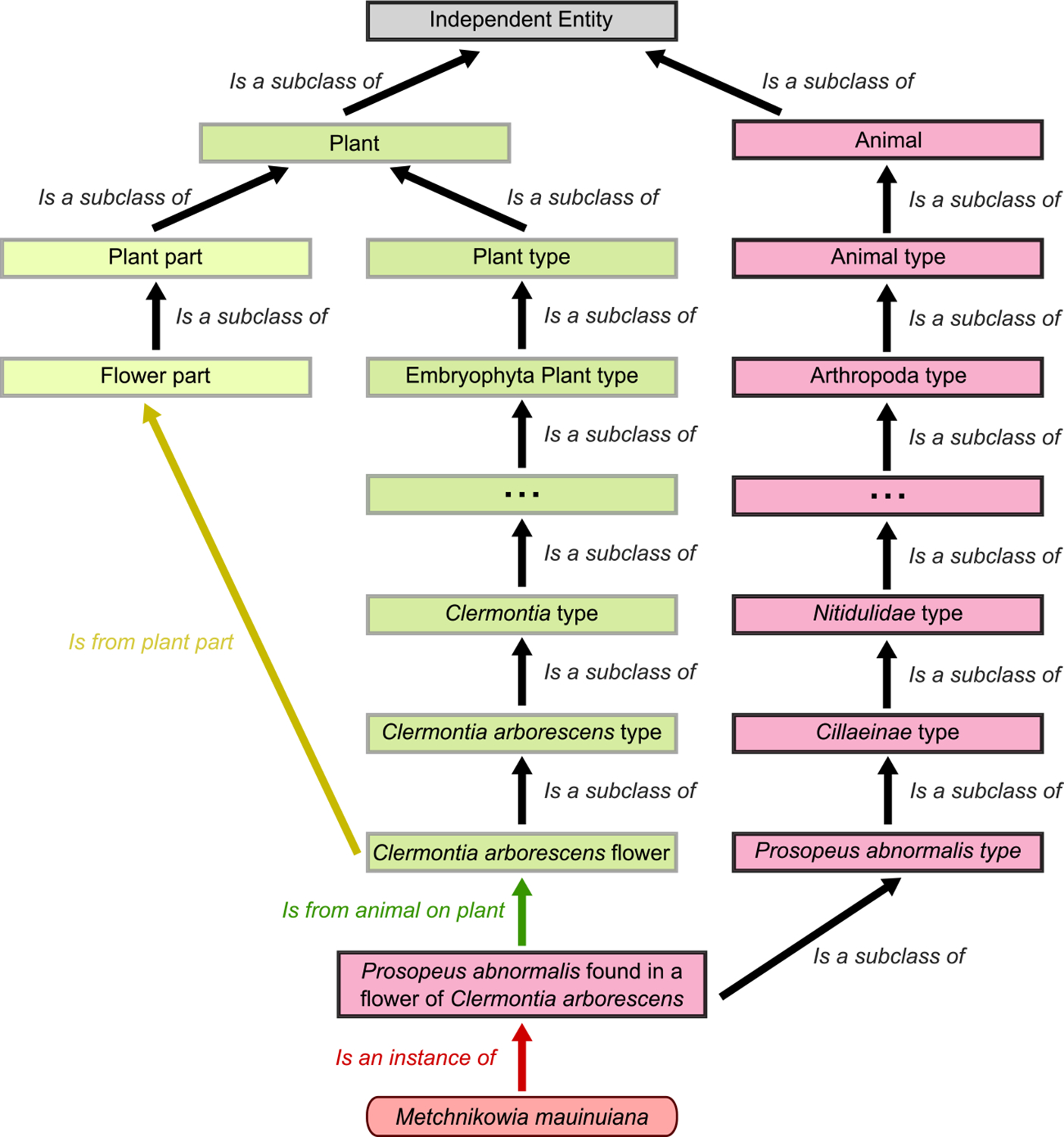
Ontology subset describing the isolation environment of *Metschnikowia mauinuiana*. Each box represents a distinct class in the ontology. Each class is a subclass of a single class higher-up in the ontology. There are two relational properties shown in the figure (green and yellow arrows) that describe relationships between classes. The strain of *M. mauinuiana* shown is an instance (red arrow) of the specific environment from which it was isolated.

In Step 2, we reviewed the isolation environment information and created the most general exclusive classes for environments—animal, plant, environmental, fungal, industrial products, and victuals (food or drink). Industrial products and victuals are composed of substrates from many origins and are differentiated by whether they are edible (victuals) or not (industrial product). We also identified subclasses within these classes, such as *type, part*, and *product* (Figure 1.) A *type* is a specific instance of the category. For example, a hexapod is a *type* of arthropod, which is a *type* of animal. A *part* is a specific region of that category, such as the intestine, which is an internal part of the animal which is a *part* of an animal. Finally, a *product* is a material that originates from the type but can be collected or separated. For example, feces are a *product* of animals.

In Step 3, we identified important features that may apply to some, but not all, of these environments, such as an association with microbes and the state of matter. These features have subcategories, such as fermented as a subcategory of microbial association. We also outlined the modifier and relational properties that connect our categories. Many secondary associations exist between categories identified in the isolation environments, such as an insect found on a specific plant. Therefore, we created relational properties, such as “is from animal on plant.” We created modifier properties, such as “has microbe association,” to identify the relationship between our categories and the features. This step allowed us to define our ontology’s scope and general structure.

Step 4 was to define the class hierarchy. We used the Web Protégé application to allow for collaborative work and visualization. The highest level of the hierarchy was split into exclusive classes: animal, plant, environmental, fungal, industrial products, and victuals. The types within animals, plants, and fungi followed generally recognized species taxonomy. For example, Diptera is a subclass of Insecta, a subclass of Hexapoda, and so on. The class hierarchy is not an exhaustive list of every known species but is based on the specific species identified in our isolation data. Due to this feature, the distances along the hierarchy are arbitrary. The high-level classes of the ontology (fungi, plants, and animals) contain a set of subclasses for parts. For example, pollen is a subclass of flower, which is a subclass of plant parts. The high-level classes defined as environmental, products, and victuals contained relevant subclasses, such as pilsner as a subclass of beer as a subclass of beverage. We exhaustively examined all the isolation environments to build our class hierarchy and relational properties. The lowest level of the hierarchy was the specific isolation environment for each yeast.

The final step, Step 5, was to create an instance of each of our yeasts in our hierarchy and assign it to the proper classes and relationships based on the description of its isolation environment. We decided the most specific class would represent the direct environment from which the yeast was isolated. For example, if a yeast was isolated from a beetle on a flower, the beetle was considered the primary or direct class. The association with flowers would be a relational property defined as “is from the animal on the plant.” The ontology contained 1,088 instances (yeasts), 1569 classes, and 27 object properties. Yeasts with detailed descriptions of their isolation environments were associated with upwards of 20 classes ranging across the hierarchy. Each yeast, however, had only one direct set of classes representing the primary environment (bold red boxes in Figure 1). Yeasts with sparse descriptions were associated with only a few classes. For example, *Lipomyces tetrasporus* is described only as being isolated from soil. Therefore, its classes are limited to soil-environment, which is a subclass of terrestrial environment, and then environmental classes. Yeasts with sparse descriptions will be limited to higher classes in the ontology and will not be included in the more specific classifications to which they may indeed belong. The classes with the most instances are those that are higher up on the ontology and include yeasts with sparse and thorough ecological descriptions.

This yeast isolation ontology was developed using Web Protégé (http://protege.stanford.edu), which is a part of the Protégé project (Musen & Protege, 2015). It is presented in the standard Web Ontology Language (OWL) file format for downstream analysis. We have also provided the OWL file for the yeast ontology as a part of the recent publication’s supplement (Opulente et al. 2024).

### 2.2 | Random Forest Construction

Random forest construction was conducted in R v4.2.2-mpi. The features used on model construction were the presence and absence (encoded as 0 and 1) of KEGG Orthologs (KOs) by KEGG obtained from previous work (Opulente et al. 2024). KEGGs with a presence below 20% across all species were removed. An initial random forest was tuned twice using the ranger package v0.16.0 (Wright & Ziegler 2015) and parsnip v1.2.1 (Kuhn & Vaughan 2024), withholding 20% of the data for validation. The first tuning was a grid search based on an initial tuning of the model. The mtry (number of variables to split at each node) and min_n (minimum number of data points for node splitting) values obtained from this tuning were then used in another grid search using 0.75 and 1.25 times the values of the first search. The final random forest model parameters were selected based on the model’s maximum area under the curve (AUC). We then constructed 100 random forest models using a different training and testing data set for each iteration. For each of the 100 random forest models, we withheld 20% of the data for model construction. The model parameters, classifications, and important features (measured by permutation in the ranger package) were stored for each iteration.

### 2.3 | KEGG Analysis

It is important to note that we filtered out results from the KEGG pathways labeled “ – yeast.” Our previous analysis (Opulente et al. 2024) showed that the KEGG database narrowly defines these as pathways in the Saccharomycetales and are under-annotated across species, especially in yeasts from other orders. We also manually re-checked KO presence and absence to verify the results of the automatic KO analysis previously conducted and removed KOs with significant differences in the re-annotation.

We analyzed KEGG orthologs that were identified in the top 1000 most important KOs in 80% of the 100 random forest models. These KOs were then run through an enrichment analysis to identify enriched pathways. This analysis was conducted in clusterProfiler v 4.10.1 (Yu et al. 2012) using the Benjamini-Hochberg multiple-testing correction. The possible universe was defined as all the KOs annotated in the input yeast genomes. Using a Fisher’s exact test, we re-analyzed each KO’s presence and absence counts across the classifications. We report the raw uncorrected p-value and odds ratio for each KEGG.

## 3 | Results

### 3.1 | Interrogation of Yeast Ecology Using the OYE

**FIGURE 2 |:**
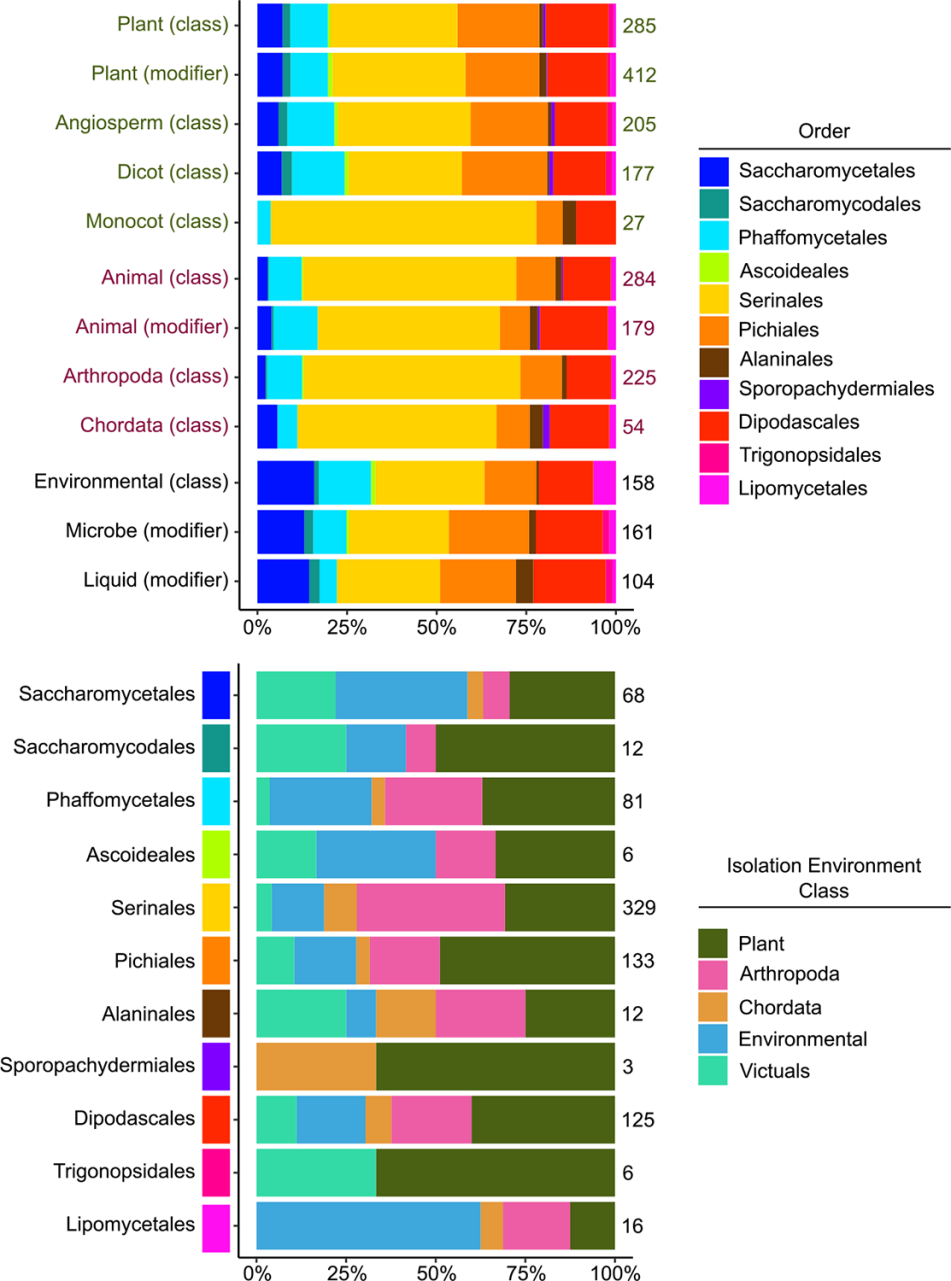
Relative distribution of the isolation environments in the ontology which includes 1088 yeasts. (A) The categories labeled “class” include yeasts that are an instance of that class or any of its subclasses. The categories labeled “modifier” are those connected to that class by a relationship. For example, any instance that contains the modifier “is from plant on animal” would be included in “Animal (modifier).” These classes are not exclusive—a yeast can be counted in both the “Plant” and “Angiosperm” categories. (B) Each order is divided into one of 5 exclusive categories, which are all classes. Therefore, no yeast is counted twice in this section. Not all yeasts, however, are classified into these groups. For example, there are 430 Serinales in this data set; due to the small overall number of samples, those sampled from other fungi are not shown.

The ontology allows us to interrogate where the 1088 yeasts were isolated from. For example, we saw a higher proportion of Pichiales yeasts in classes associated with the plants class (65/285: 23%) than with the animals class (31/284: 11% Figure 2A). We also interrogated which environments were predominant within each recently established yeast order (Groenewald et al. 2023). The majority of Lipomycetales yeasts were isolated from the environment class (10/16: 63%), and almost half of the Serinales were isolated from the Arthropoda class (136/329: 41%; Figure 2B). The ontology can also be interrogated at much more refined levels. There were 124 classes that contained between five and ten instances. There were five yeasts that were isolated from mushroom fruiting bodies: *Candida inulinophila, Candida morakotiae*, *Candida smagusa*, *Kodamaea fukazawae*, and *Kodamaea fungicola*, which all belong to the order Serinales. There were 6 yeasts isolated from cows: *Nakazawaea peltata* (Alaninales), *Kockiozyma suomiensis* (Lipomycetales), *Wick-erhamomyces bovis* (Phaffomycetales), *Magnusiomyces capitatus*, *Yarrowia hollandica*, and *Zygoascus hellenicus* (Dipodascales).

### 3.2 | Classification of Yeasts Isolated From Plants and Animals Using Genomic Data

**FIGURE 3 |:**
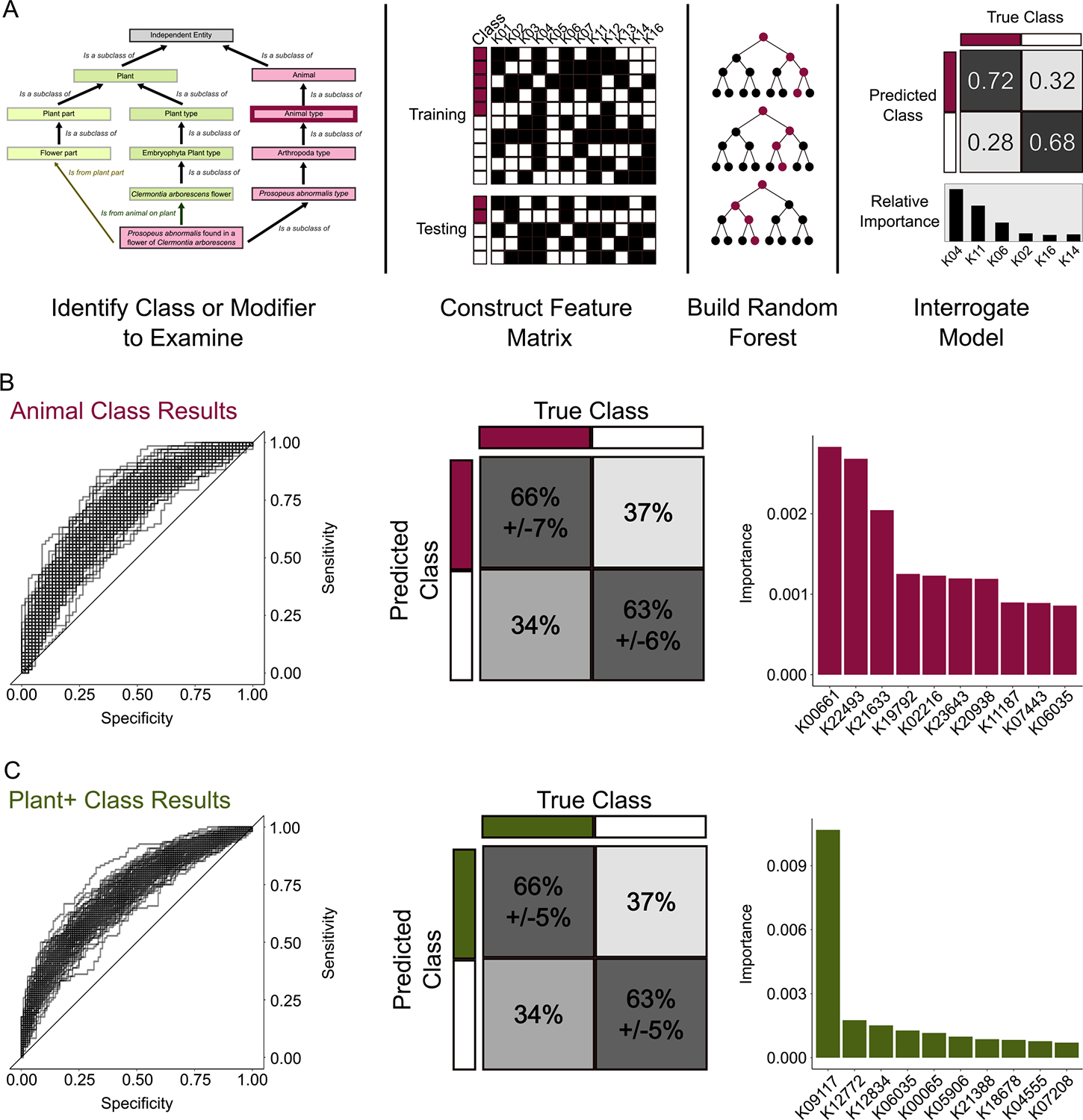
The Ontology of Yeast Environments enabled machine learning analysis identify genes associated with specific environments. (A) The general framework for utilizing the yeast ecological ontology for machine learning. We identified a specific class of interested and obtained all the instances (yeast strains) either directly (black arrows) or relationally (colored arrows) associated with that class. The instances were then divided into training and testing datasets where the presence and absence of KEGG Orthologs (KOs) were used as features. We constructed a random forest and then interrogated the model for accuracy and the important features. (B) Classification of yeast in the animal class had an average AUC of 0.71 and an average true-positive rate of 66% across 100 iterations of the model. The KOs with the highest permutation importance are shown in the bar graph. (C) Classification of yeast in the plant class (including relational associated but with decayed plants removed) had an average AUC of 0.71 and an average true positive rate of 66% across 100 iterations of the model. There was a single KO (K09117) that had three times higher importance as the next most important KO.

To further demonstrate the utility of the ontology, we conducted a machine learning analysis aimed at identifying genes or pathways associated with specific classes in our ontology. We trained a random forest algorithm using the R programming language to classify yeasts as present or absent in each of the ontology classes (Figure 3A.) The binary data matrix generated from the ontology differentiated between direct subclassifications and the relational values between (black lines vs. colored lines in Figure 1.) The features used to train the model were the predicted presence or absence of genes identified by the Kyoto Encyclopedia of Genes and Genomes (KEGG), which was previously generated across all 1154 yeasts (Opulente et al. 2024). Briefly, the random forest parameters were tuned to maximize the accuracy and precision of the model. These parameters were then used to train a random forest model using a balanced data set where 20% of the data was withheld for testing, and 80% was used for training. Models that classified yeasts better than random were then further interrogated by repeating the random forest construction 100 times to examine the impact of the training data set. The code and complete results can be found in the FigShare repository.

The two most successful models (Figure 3B,C) were able to classify yeasts into the class plant (mean AUC of 0.67) or class animal (mean AUC of 0.71). AUC is the area under the receiver operating characteristic curve (ROC), which compares accuracy and precision. Accuracy is a measure of the overall classification success and precision is a measure of per-class success. Therefore, we can classify the isolation environments of yeasts isolated from plants and animals much better than random from gene presence/absence data. The success of these two specific categories is likely related to their large sample sizes (366 for plant and 339 for animal). We then investigated the yeasts that were consistently misclassified (false positives and false negatives) by the algorithm (FigShare Repository.) We noted that a substantial number of yeasts (284 yeasts) were falsely classified as belonging to the plant class if they were isolated from insects associated with plants. Additionally, many yeasts (109) isolated from decaying or dead plants were falsely classified as not associated with plants. We, therefore, reconstructed the model to classify yeasts as belonging to a plant class or having the relational value “from plant” but not the relational value “decayed microbe association.” For example, in the original model, *Metschnikowia shivogae*, which was isolated from “insects of morning glories,” was not included as an instance of plants. Due to the secondary association, *M. shivogae* was changed to a positive instance in the new model. Conversely, *Sugiyamaella lignohabitans*, which was isolated from “decayed wood” was initially included as a positive instance of plants but was subsequently changed to a negative instance due to its association with decay. When these adjustments were made, the model performance improved from a mean AUC of 0.67 to 0.71. Using the ontology allowed us to easily adjust our data to capture various aspects of the association between yeasts and plants.

For each final model, we also investigated which yeasts were consistently misclassified (full data in the FigShare repository). For example, in the animal model, 33 yeasts were falsely classified as not animal-associated in every iteration of the model. We could not, however, identify a specific pattern in this group as the isolation environments ranged from the gut of a histerid beetle (*Dipodascus histeridarus*) to the blood of a mink (*Candida blankii*). Conversely, there were 100 yeasts consistently falsely classified as animal-associated ranging in isolation environment from mangrove forest water (*Candida nonsorbophila*) to sake-moto (*Candida sake*).

### 3.3 | Genes and Pathways Enriched in Animal-Associated Yeasts

**FIGURE 4 |:**
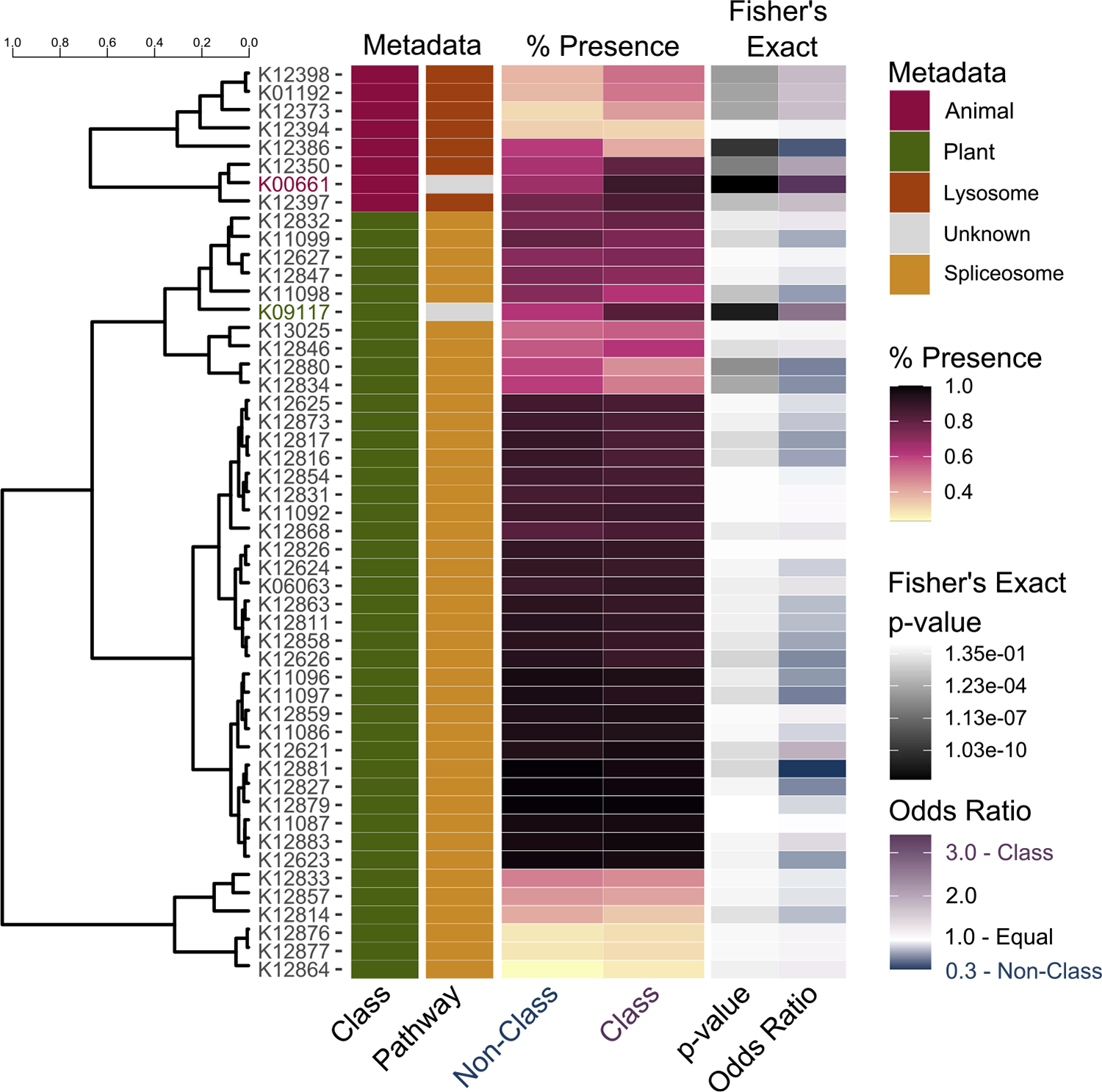
KOs with known and unknown functions were highly informative in the construction of the random forest to classify yeast as isolated from plants or animals. The KOs associated with classification of yeasts in the animal or plant classes (first column) were clustered according to presence in the analyzed class (% Presence columns). The associated pathway for each KO is shown in column 2 with the two most important KOs (colored names) belonging to no known pathway. We also tested for statistical differences in the presence of the KOs in the yeasts belonging to the examined class as compared to those not in that class using a Fisher’s exact test. The p-value and odds ratio are reported in the last two columns and the raw data is presented in the FigShare repository.

We interrogated the KEGG genes that had the highest median permutation importance across the iterations of the models (Figure 4). This analysis allowed us to ask which genes or pathways are important for classifying yeasts as associated with animals. In every model iteration, the KEGG ortholog (KO) K00661 was in the top 1000 most important features and had the highest median importance (0.0028). This KO encodes a maltose O-acetyltransferase and is annotated in the *S. cerevisiae* genome as an uncharacterized ORF with the systematic name YJL218W. In yeasts isolated from animals, 87% (295/339) have a copy of this gene compared to only 66% (495/747) of nonanimal yeasts. Previous work has shown that Oaf1p/Pip2p induces this gene in *S. cerevisiae* in the presence of oleate (Smith et al. 2002). In turn, these regulatory genes (*OAF1/PIP2*) are required for peroxisome proliferation in response to oleate, and their deletion prevents the use of oleate as a singular carBon source (Rottensteiner et al. 2003). Moreover, the YJL218W deletion strain of *S. cerevisiae* had decreased cell membrane integrity and reduced capacity to grow in high salt concentrations (Li et al. 2022). In a general framework, the presence of K00661 may improve yeasts’ abiLity to respond to stressors of the animal environment, especially increased salt concentrations (Manzanares-Estreder et al. 2017). The yeasts examined have been isolated from high salt environments like human blood (*Candida pseudoaaseri*). The Na^+^ salt concentration of insect hemolymph can reach 118 mmol/L (Natochin & Parnova 1987), while normal human blood sodium levels are ~140 mmol/L (Li et al. 2016). More specifically, the exterior and interior of insects have lipids, including oleic acid, that can both stimulate and prevent fungal growth (Keyhani 2018). Yeasts associated with insects comprise most of the animal-associated yeasts in our data set (254/339.) Of the 254 insect-associated yeasts, 227 (89%) have a copy of K00661. In addition to a general role in stress response, genes belonging to the KO K00661 may facilitate growth on and in insects.

We also examined the pathways enriched with genes important for classifying yeasts as animal associated. To identify these pathways, we conducted a KEGG enrichment using the 209 KOs identified in the feature importance analysis of our model. The lysosome pathway (ko04142) was the most highly enriched for KOs identified with our model (seven KOs), although it did not pass statistical significance (adjusted *p*-value 0.1). This pathway generally corresponds to the function of vacuoles in yeasts as they do not contain lysosomes. The important features of our model had vacuole-associated functions in enzyme transport (K12398, K12397, K12394), acid hydrolases (K12373, K01192, K12350), and membrane proteins (K12386). Two of these KOs (K12386 and K12394) had a lower abundance in animal-associated yeasts. Animal-associated yeasts are enriched in K12397 and K12398, which are both subunits of the AP-3 complex. K12397 is the β-subunit (Apl6p in *S. cerevisiae*), and K12398 is the μ-subunit (Apm3p in *S. cerevisiae*.) The AP-3 complex is involved in the selective transport of proteins from the Golgi to the vacuole (Cowles et al. 1997). The proteins transported by the AP-3 complex in *S. cerevisiae* are alkaline phosphatases (Cowles et al. 1997), a t-SNARE Vam3p (Cowles et al. 1997), yeast casein kinase 3 (Yck3p) (Sun et al. 2004), and the Niemann-Pick Type C homolog Ncr1p (Berger et al. 2007). Recent work has linked AP-3 with stress-induced vacuole fusion mediated by the protein Yck3p (44, 45) and cell death in both *S. cerevisiae* and the human pathogenic Basidiomycetous yeast *Cryptococcus neoformans* (Stolp et al. 2022). The authors who uncovered the association between AP-3 and cell death suggest that differential regulation of AP-3 may be important for human fungal pathogens (Stolp et al. 2022), which are generally included as animal-associated in our data set. Interestingly, in our data set, only K12397 is elevated in human fungal pathogens (10/11) as opposed to their relatives (49/60), according to designations from our previous work (Opulente et al. 2024). Both KOs also have a higher abundance in insect-associated yeasts (50% vs. 40% for K12398 and 82% vs. 77% for K12397).

Three acid hydrolases were also enriched in our models’ important features. These are; K12350, a sphingomyelin phosphodiesterase (Ppn1p in *S. cerevisiae*); K12373, a β-*N*-hexosaminidase (Hex1p in *C. albicans*); and, K01192, a β-mannosidase (orf19.2838 in *C. albicans*). When transported via vacuoles to the cell exterior, these proteins may break down or modify the environment to allow yeasts to obtain nutrients or combat stressors. Ppn1p cleaves polyphosphates, potentially allowing the use of polyphosphates (45) for protection from oxidative stress (46), formation of canals in the cell wall (47), or as an energy source (Rao et al. 2009). Hex1p is involved in utilizing amino-sugars, such as *N*-acetyl-d-glucosamine (GlcNAc). In *C. albicans*, this gene is critical for full virulence (Jenkinson & Shepherd 1987) and plays a role in carbon and nitrogen scavenging during infection of mouse kidneys (Ruhela et al. 2015). Finally, the β-mannosidase has been shown to impact sensitivity to amphotericin B (Xu et al. 2007) and is associated with biofilm production (Bonhomme et al. 2011). We have also found K01192 to be associated with carbon generalism in yeasts (Opulente et al. 2024).

Our analyses illustrate how the availability of a subphylum-wide yeast environment bio-ontology can be employed to identify candidate genes and pathways that may be involved in the adaptation of yeast species to animal environments. Most animal-associated yeasts are associated with arthropods (254/339), while 74 of the remaining yeasts are associated with chordates. Yeasts that are directly associated with animals and arthropod environments experience many of the same stressors, including immune cells, oxidative stress, high salinity, nutrient availability, and even temperature stress as global temperatures rise. The oleate metabolism and vacuole-associated acid hydrolase genes we identified here may be important for the adaptation to these shared stressors.

### 3.4 | Genes and Pathways Enriched in Plant-Associated Yeasts

Finally, we interrogated the model that classified yeasts as plant-associated, including those with secondary modifier associations but no association with decay (Figure 4). The KEGG with the highest consistent importance in the model was K09117. This is an uncharacterized protein known as Aim41p in *S. cerevisiae*. This gene was found in 83% (364/439) of yeasts associated with plants as compared to 61% (245/404) from non-plant environments. Previous work associated this gene with mitochondrial inheritance (Hess et al. 2009) and upregulation in stress-resistant cells found in the upper level of yeast colonies (Čáp et al. 2012). This gene is overexpressed in *S. cerevisiae* under oxidative stress when exposed to cocoa powder extract (Peláez-Soto et al. 2020). Other work has shown that the allele-specific expression of AIM41 is involved in the differential thermal tolerance of *S. cerevisiae* and *S. uvarum* (Li & Fay 2017). Recently, thermotolerance, but not this specific KEGG, has been implicated in the evolution of yeasts associated with cacti (Goncalves et al. 2023). These results suggest that plant-associated yeasts may be able to better respond to the stressors of the plant environment, such as high temperature due to solar exposure and oxidative stress in plants (Hasanuzzaman & Fujita 2022).

The spliceosome was the only pathway statistically enriched in the KEGGs important for classifying plant-associated yeasts. Forty-one KEGGs associated with the spliceosome were also associated with isolation from plants. The KEGG with the highest importance involved in the spliceosome was K12834 (median importance 0.0015), a PHD finger-like domain-containing protein 5A and known as Rds3p in *S. cerevisiae*. This KEGG is absent in 51% (281/550) of the plant-associated yeasts and 39% (211/536) in the non-plant-associated. Despite high conservation in the spliceosome of eukaryotes, previous work in yeasts has shown high variability in the spliceosome, which is likely associated with the loss of introns across the group (Bon, 2003). Alterations in the major components of the spliceosome, especially in U4/U5/U6 tri-snRNP, have been shown in yeasts during heat stress response (Bond 2006; Bracken and Bond 1999). Two components of the U4/U5/U6 tri-snRNP were important in our model; these were the SM (SNRPB/D2/E/F/G) and LSM (Like Sm; including LSM2/4/5/6/7/8) proteins. We hypothesize, therefore, that the presence and absence of specific spliceosome components may increase or decrease a yeast’s ability to respond to specific stressors.

Yeasts associated with the plant or plant-insect environment have a distinct set of important features when compared to animal-associated yeasts. This suggests that the stressors of the plant-insect environment are also distinct. The exact stressors that Aim41p and the spliceosome respond to in the plant environment are not fully elucidated, but both pathways have been associated with heat tolerance.

## 4 | Future Perspectives

The OYE allowed us to transform individual yeast species descriptions written in natural language into a format interpretable to machine learning algorithms, enabling subphylum-level systematic analyses of yeast isolation environments. By training our machine learning model using gene presence and absence features, we could classify yeasts into those isolated from animals and those isolated from plant or plant-associated environments. Given that yeasts are likely to be found in multiple environments and that adaptation to these environments is likely highly pleiotropic, it is remarkable that our model reaches an accuracy better than random. In our data set, we were able to uncover novel associations between genes or pathways and yeasts that were isolated from specific environments.

The associations we identified require follow-up testing to fully interrogate the role of these genes in adaptation to environments. Nevertheless, we can formulate testable hypotheses from our analysis. For example, in both plant- and animal-associated yeasts we identified different sets of genes with previously reported roles in stress response. Interestingly, there are known parallels between human and plant pathogenesis in fungi (Sexton & Howlett 2006). We could, therefore, test to see if yeasts that contain both the plant-associated and animal-associated genes are more likely to colonize both types of tissue.

The ontology and machine-learning analysis have some limitations. Due to sample size constraints, we focused primarily on the highest-level classes of the ontology in this analysis. This level of classification may lump together yeasts from disparate environments (insect vs. mammal in the animal class) and obscure more specific gene-environment associations. Increasing the number of yeasts classified will address this limitation. The random forest model also has some limitations such as that it requires exclusive classifications—a yeast cannot belong to two classes simultaneously. The application of more complex models that can directly infer the ontological structure may improve our ability to interrogate the data.

We anticipate that this ontological framework for isolation environments will be foundational and enable computational complex analysis of wide-ranging yeast ecological data. When DNA is collected from an environment, the metadata often includes natural language descriptors similar to species descriptions. For example, metagenomic samples have recently been collected from soybean rhizosphere (MGNify MGYS00006228) and a whale’s blow hole (MGNify MGYS00006536). While natural language interpretation of these environments allows us to know that they are very different, downstream data analysis will require a framework, such as an ontology.

The OYE was created with the explicit purpose of interrogating strain-specific variation in isolation environments associated with the Y1000+ Project genomes (Opulente et al. 2024). To improve the breadth of the ontology, the Y1000+ Project is also adding additional strains for the species sequenced in the project. While we believe that this ontology serves as a foundational resource, maintaining and expanding it to capture all of yeast diversity would require a substantial commitment from yeast researchers and culture collections. Therefore, the OYE created here can serve as a model upon which a universal yeast environment ontology could be created. Alternatively, researchers can adapt the OYE to suit their individual needs.

Our ability to connect yeast traits to their environments is only as good as our environmental data. An ontology allows us to capture many aspects of yeast environments in a format that enables the use of powerful machine-learning algorithms. The ontology is also adaptable to historical natural language descriptions and modern metadata collection. Just as phylogenies have enabled investigation of the history of the yeast subphylum, a formalized ontology could transform the way we study the role of environment in yeast function and evolution.

## Data Availability

The Y1000+ data can be obtained from the project website (http://y1000plus.org). The Figshare repository https://figshare.com/projects/Exploring_Saccharomycotina_Yeast_Ecology_Through_an_Ecological_Ontology_Framework/208648 the raw random forest model data and a copy of the ontology.

## Outstanding Questions

- The construction of the environmental ontology relies heavily on the natural language descriptions recorded during strain or metagenomic sampling. How can we adapt standards that improve the detail in these descriptions to better capture primary and secondary associations?
- How can we integrate the rapidly growing body of genomic, ecological, and phenotypic data to identify yeast adaptations in response to specific environmental niches?
- Can we integrate the environmental and metagenomic data with our ecological ontology to compare across environments?

## Summary

- Ontological frameworks allow high throughput analysis of ecological data.
- We established a formal Ontology of Yeast Environments.
- The Ontology of Yeast Environments describes isolation environments for 1088 strains.
- Coupled with genomic data, analysis of the ontology reveals gene-environment associations.
